# Metabolomic Characterization and Utilization Assessment of Kumquat Germplasms

**DOI:** 10.3390/foods15213978

**Published:** 2026-11-05

**Authors:** Changwei Zhu, Liu Yang, Haole Shang, Shuai Cao, Hongmei Huang

**Affiliations:** Hunan Engineering Laboratory of Miscanthus Ecological Applications, College of Bioscience and Biotechnology, Hunan Agricultural University, Changsha 410128, China; wei202610@163.com (C.Z.);

**Keywords:** kumquat, metabolomics, metabolic pathways, flavonoids

## Abstract

Kumquat (*Fortunella*), as a fruit with both medicinal and edible properties, contains various bioactive components and possesses multiple functional activities. In this study, 13 kumquat germplasms were used as experimental materials, and untargeted metabolomics and flavonoid-targeted metabolomics were employed to investigate the metabolic basis and differences underlying fruit quality variations among different kumquat germplasms. The results revealed significant metabolic differences between *F. crassifolia* and other germplasms: compared with *F. crassifolia*, *F. hindsii* exhibited higher Salicin content and was rich in flavonoids; *F. obovata* showed significant differences in the nicotinate and nicotinamide metabolic pathways; and the Cuipi germplasm had lower organic acid content. These results indicate that Cuipi is most suitable for fresh consumption, *F. crassifolia* is suitable for both fresh consumption and medicinal use, *F. hindsii* has great potential for the medicinal development of Salicin and flavonoids, and the other germplasms are suitable for processing, medicinal use, or ornamental purposes.

## 1. Introduction

Kumquat (*Fortunella*) is an evergreen plant belonging to the Rutaceae family. It is native to southeastern China and was introduced to Europe in the mid-19th century, subsequently spreading to the Americas. Today, it is cultivated in many countries and regions, including the United States, Brazil, Japan, India, Australia, and Colombia, among others [1]. Kumquat has the highest edible rate among citrus fruits, with the edible rate of Rong’ancuimi reaching 98.40% [2].

Kumquat is rich not only in primary metabolites such as sugars, organic acids, and amino acids, but also in secondary metabolites including flavonoids, phenolic acids, and volatile components [3,4,5]. Integrating primary and secondary metabolites holds significant practical implications for germplasm selection and food processing. The composition of primary metabolites directly determines the fresh-eating flavor (e.g., sugar-to-acid ratio) and fundamental processing characteristics of the fruit, whereas secondary metabolites not only confer pharmacological activities such as antioxidant and anti-inflammatory effects but also influence the formation of specific flavors (e.g., bitterness and astringency) and color. So these metabolites not only contribute to the diverse fruit flavors of kumquat but also confer potential therapeutic effects in diseases such as cancer [6,7] and diabetes with hyperlipidemia [8,9], indicating its great potential for the development of medicinal functional components. Using antioxidant evaluation methods such as 1,1-Diphenyl-2-picrylhydrazyl (DPPH) and 2,2′-Azino-bis(3-ethylbenzothiazoline-6-sulfonic acid) (ABTS), researchers have found that flavonoids, volatile compounds like α-pinene and limonene, as well as polysaccharides, all possess free radical scavenging capacity, thereby exerting antioxidant effects [10,11]. Furthermore, studies have reported that metabolites in kumquat exhibit excellent inhibitory effects against *Staphylococcus aureus* and *Bacillus subtilis* [12,13].

With the rapid development of high-throughput analytical technologies, metabolomics has become a powerful tool for comprehensively and systematically revealing the associations between plant genetic diversity and metabolic phenotypes. It also enables the qualitative and quantitative analysis of metabolites, thereby providing guidance for the utilization of plants. Guo et al. [14] used ultra-performance liquid chromatography coupled with triple quadrupole tandem mass spectrometry(UPLC-QqQ-MS/MS) to detect and analyze flavonoids, phenolic acids, limonoids, alkaloids, and coumarins in six kumquat species, including Wenzhouluofu (*F. margarita*), Ronganjindan (*F. crassifolia*), and Ningboluowen (*F. japonica*). Their results showed significant differences in the contents of these metabolites among the six species. Goh et al. [15] analyzed volatile metabolites in four kumquat species and found that limonene was the most abundant volatile metabolite in all varieties, whereas the contents of other metabolites such as palmitic acid, linalool, and germacrene D varied among germplasms, with Huapijingan having particularly high linalool content. In addition, comparative studies on metabolite differences between Huapijingan and Rong’anjingan are abundant. Ma et al. [16] found that the total flavonoid content in Huapijingan fruits was more than four times that in Rong’anjingan, with hexosylated flavonoids being particularly prominent, while the lipid content was relatively lower. Huang et al. [17] reported that the wax content in the peel of Huapijingan was higher than that in Rong’anjingan, which is beneficial for postharvest storage. He et al. [18] found that the organic acid content in Huapijingan was lower than that in Rong’anjingan and further analyzed the mechanism of citric acid accumulation in the two cultivars. The above findings indicate that the types and contents of metabolites in the fruits of different kumquat germplasms differ significantly.

As a fruit with both medicinal and edible properties, kumquat contains various bioactive components and exhibits multiple functional activities. However, most existing studies are limited to the analysis of metabolites and partial functional activities in only a few germplasms, and the research on metabolite differences among different kumquat germplasms is insufficiently systematic and comprehensive. Consequently, it is not possible to achieve precise processing and utilization based on these metabolite differences. Therefore, this study aims to conduct a comparative analysis of differential metabolites among different kumquat germplasms using metabolomics approaches, elucidate the accumulation differences of 24 flavonoids in five kumquat cultivars, obtain comprehensive metabolic information of kumquat germplasms, and explore the metabolic basis underlying the differences in fruit quality among various kumquat germplasms, thereby providing a theoretical foundation for the processing, utilization, and variety breeding of kumquat.

## 2. Materials and Methods

### 2.1. Plant Materials

In this study, 13 of the most widely cultivated and representative kumquat germplasms in China were selected from the Kumquat Germplasm Resource Nursery of Hunan Agricultural University, representing five kumquat species: *F. crassifolia*, *F. hindsii*, *F. margarita*, *F. obovata*, and *F. japonica*. All trees of these germplasms have been cultivated and managed uniformly in the resource nursery for ten years. Since the pericarps of *F. crassifolia* No. 11 and No. 12 lack oil glands, they were separated into a sixth group, hereafter referred to as “Cuipi”. In the planting area of each kumquat germplasm, 5–6 trees were selected using a “Z”-shaped sampling method. A total of 16 fully ripe fruits, free of pests and diseases, uniform in size, and undamaged, were harvested from each tree in four directions (east, south, west, and north). The fruits were brought back to the laboratory, vacuum freeze-dried, and ground into a dry powder. Samples of the same germplasm were subjected to “quartering” for sampling, passed through a 40-mesh sieve, and then sealed and stored in a low-temperature freezer in the dark. Detailed sample information is provided in Table 1.

### 2.2. Non-Targeted Metabolomics Analysis of Secondary Metabolites in Different Kumquat Germplasms

Accurately weigh 0.05 g of the previously prepared kumquat dry powder into a 2 mL Eppendorf (EP) tube, accurately add 1.5 mL of 70% methanol (HPLC grade, Merck, Darmstadt, Germany), and shake well. Perform ultrasonic extraction for 40 min at 25 °C and 40 kHz, followed by centrifugation at 10,000 rpm for 10 min. Transfer the supernatant to a new 2 mL EP tube. The extract was filtered through a 0.22 μm organic phase syringe filter, transferred into a sample vial, and vortexed for 30 s before instrumental analysis. Three subsamples were independently weighed from the pooled powder of each germplasm, serving as three biological replicates, and data were acquired in both positive and negative ion modes. Quality control (QC) samples were prepared by pooling equal volumes of the extract from each sample. During the ultra-high-performance liquid chromatography coupled with quadrupole time-of-flight mass spectrometry (UPLC-Q-TOF-MS) analysis, a QC sample was injected every eight samples to monitor method stability and data reliability.

Non-targeted metabolomics analysis was performed using ultra-performance liquid chromatography coupled with UPLC-Q-TOF-MS. The liquid chromatography analysis was conducted on a Shimadzu Nexera X2 UPLC system (Shimadzu, Kyoto, Japan) equipped with a Waters HSS T3 column (1.8 μm, 2.1 mm × 100 mm). The injection volume was 5.0 μL, with a flow rate of 0.3 mL/min and a column temperature of 45 °C. The mobile phase consisted of (A) water containing 0.1% formic acid and (B) acetonitrile containing 0.1% formic acid. The gradient elution program was as follows: 0–5 min, 2% B; 5–7 min, 2–13% B; 7–21 min, 13–21% B; 21–23 min, 21–30% B; 23–28 min, 30–100% B; and 28–30 min, 100% B.

Mass spectrometry analysis was performed on a Triple TOF 5600 mass spectrometer (AB SCIEX, Framingham, MA, USA) using the data-independent acquisition (DIA) mode. The TOF-MS scan range was set to m/z 100–800, while the TOF-MS/MS scan range was m/z 50–1200. An electrospray ionization (Turbo spray) source was employed, with scanning performed in both positive (ESI+) and negative (ESI−) ion modes. In positive ion mode, the spray voltage was 5.5 kV and the orifice voltage was 80 V; in negative ion mode, the spray voltage was −4.5 kV and the orifice voltage was −80 V. The nebulizer gas, auxiliary gas, and curtain gas were set to 50 L/min, 50 L/min, and 35 L/min, respectively. The collision energy was 35 eV and −35 eV, with a collision energy spread of 15 eV. The accumulation times for TOF-MS and TOF-MS/MS were both 150 ms. Prior to each analysis, an automatic calibration delivery system was used for precise mass calibration.

Metabolite identification was confirmed by matching the acquired detection data against the mzCloud, mzVault, Human Metabolome Database (HMDB), and Kyoto Encyclopedia of Genes and Genomes (KEGG) databases. The identification results were annotated according to the metabolite identification levels defined by the Metabolomics Standards Initiative (MSI): Level 1, metabolites in the samples matched the MS1, MS2, and retention time (RT) of authentic standards; Level 2, metabolites in the samples matched the MS1 and MS2 of public databases; Level 3, metabolites in the samples matched the MS1, MS2, and predicted RT of theoretical databases; and Level 4, unknown compounds.

Prior to statistical analysis, the raw metabolomic data were preprocessed. Features with missing values exceeding 50% in any group were removed, and the remaining missing values were imputed using the mean value. Subsequently, the data were Log2-transformed and normalized using total ion current (TIC) to minimize systematic bias. After peak alignment, peak area extraction, retention time correction, and further data preprocessing, the metabolite data from Levels 1 and 2 were retained and converted into a data matrix. This matrix included datasets such as retention time, mass-to-charge ratio (m/z), compound name and classification, chemical formula, and corresponding ion intensity.

Multivariate statistical analysis was performed using SIMCA 14.1 software(Umetrics, Umeå, Sweden). Univariate analysis was conducted using independent samples t-tests. The Benjamini–Hochberg method was applied to control the false discovery rate (FDR) for multiple testing correction, and the effect size was represented by fold change (FC). Orthogonal partial least squares discriminant analysis (OPLS-DA) was used for multivariate statistical modeling, and variable importance in projection (VIP) values were calculated. The screening criteria for differential metabolites were: VIP > 1, FC > 2, and adjusted *p*-value (FDR) < 0.05. Finally, the differential metabolites were uploaded to the Kyoto Encyclopedia of Genes and Genomes (KEGG) database for metabolic pathway annotation analysis [19].

### 2.3. Targeted Metabolomics Analysis of Flavonoids in Different Kumquat Germplasms

To ensure representativeness, five kumquat germplasms—Liuyangjindan (*F. crassifolia*), NingboluowenCQ (*F. japonica*), Xiaoguojindou (*F. hindsii*), Wenzhouluofu (*F. margarita*), and Shouxingju (*F. obovata*)—were selected from the five respective species for targeted flavonoid metabolomics analysis. Accurately weigh 100 mg of vacuum freeze-dried kumquat powder and transfer it into an EP tube. Add 1 mL of 70% methanol–water (pre-cooled at −20 °C) to each tube. After vortexing, the 2 mL grinding tube was transferred and ground at 60 Hz for 90 s. Allow the samples to fully extract by incubating on ice for 30 min. Subsequently, centrifuge at 12,000 r/min for 3 min at 4 °C. Transfer 300 μL of supernatant to a new EP tube, dry using a vacuum centrifugal concentrator, and then redissolve in 50 μL of methanol/water (7/3, *v*/*v*). After centrifugation, collect 30 μL of supernatant for instrumental analysis. Biological replicates were prepared following the same procedure as described in Section 2.2.

Chromatographic separation of target compounds was performed using a Thermo Scientific Vanquish Core HPLC system equipped with a CSH C18 column (50 mm × 2.1 mm; 1.7 μm, Waters). The mobile phase consisted of (A) 10 mM ammonium acetate in water and (B) acetonitrile. The column temperature was maintained at 40 °C, the sample tray was set at 15 °C, and the injection volume was 1 μL.

Mass spectrometric analysis was conducted using a Thermo Fisher Q Exactive HF-X MS Orbitrap mass spectrometer (Thermo Fisher Scientific, Bremen, Germany) with the following ion source parameters: spray voltage 3200 V, capillary temperature 320 °C, sheath gas flow rate 30 arb, auxiliary gas flow rate 10 arb, and auxiliary gas heater temperature 400 °C.

All target compound data analysis for qualitative and quantitative purposes was performed using Thermo Xcalibur Qual Browser software for data acquisition and processing.

## 3. Results

### 3.1. Analysis of Non-Targeted Metabolomics Detection Results of Secondary Metabolites in Different Kumquat Germplasms

After preprocessing, for kumquat samples of different germplasms, and after removing potential false-positive metabolites, a total of 3192 metabolites were successfully identified in positive ion mode, and 2185 metabolites were identified in negative ion mode, providing quantitative information for a total of 5377 metabolites. These essentially cover flavonoids, limonoids, organic acids, phenolic acids, volatile components, coumarins, and other substances. Overall, more metabolites were detected in positive ion mode than in negative ion mode.

### 3.2. PCA of Metabolites in Different Kumquat Germplasms

**Figure 1:**
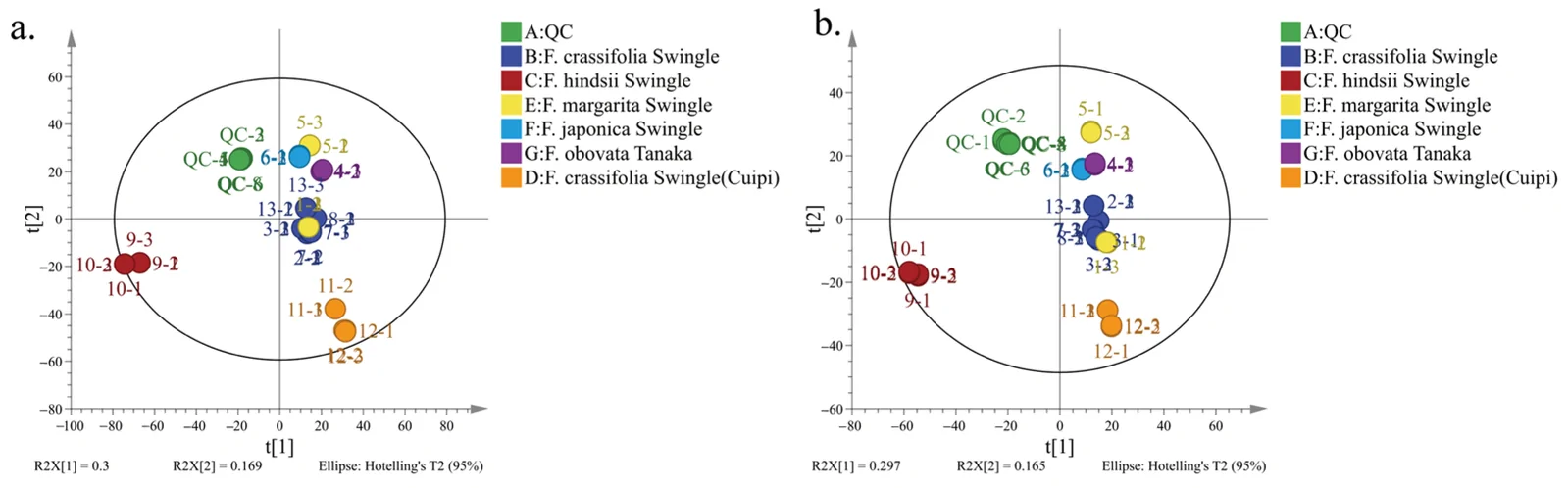
PCA plots of positive ion mode (**a**) and negative ion mode (**b**) of different kumquat germplasm metabolites.

Firstly, we used principal component analysis to analyze the overall differences in metabolites in different kumquat germplasms. In the positive ion mode (Figure 1a), a total of 14 principal components were extracted from the PCA model, and the fitting parameters were R^2^X = 0.991 and Q^2^ = 0.97. In the negative ion mode (Figure 1b), a total of 15 principal components were extracted from the PCA model, and the fitting parameters were R^2^X = 0.993 and Q^2^ =0.972, indicating that the model had a good fitting effect and could be used for subsequent analysis. Quality control samples clustered near the center of the PCA plot, and all samples fell within the 95% confidence interval, confirming data reliability.

Notably, the *F. hindsii* group exhibited clear separation from other samples, suggesting distinct metabolic profiles. This divergence may reflect its status as the earliest evolved species within the *Fortunella* genus. In contrast, close proximity was observed between *F. margarita* and *F. crassifolia* samples, with overlapping data points indicating metabolic similarity. These patterns provide a foundation for subsequent differential metabolite analysis.

### 3.3. OPLS-DA Analysis of Metabolites in Different Kumquat Germplasms

**Figure 2:**
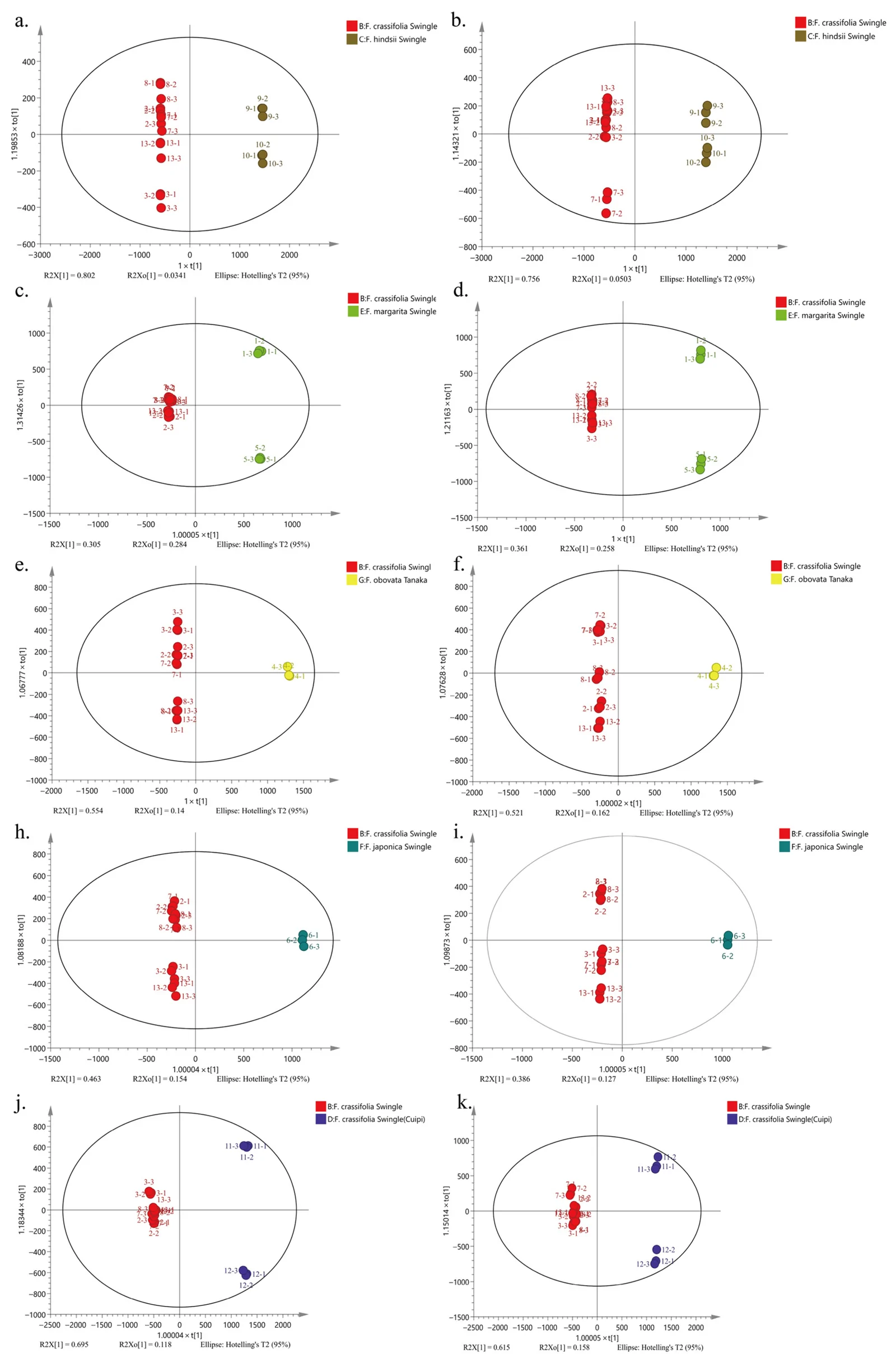
Plot of OPLS-DA scores of positive ion mode (**a**,**c**,**e**,**h**,**j**) and negative ion mode (**b**,**d**,**f**,**i**,**k**) for the *F. crassifolia* group and other germplasms.

In order to observe the differences between different kumquat germplasms more intuitively, we used the *F. crassifolia* Swingle group as the control group and compared it with the *F. hindsii* Swingle group, the *F. margarita* Swingle group, the *F. obovata* Tanaka group, the *F. japonica* Swingle group, and the *F. crassifolia* Swingle (Cuipi) group in the positive and negative ion modes. The pairwise comparison of the orthogonal partial least squares discriminant analysis (OPLS-DA) model (Figure 2) was carried out for pairwise comparison. The fruit sample points of each group were distributed in different regions and were clearly divided into different clusters. All samples were within the 95% confidence interval, indicating that there were significant differences in the types and quantities of differential metabolites in each group.

As shown in Table 2, the model statistical parameters R^2^Y (cum) and Q^2^ (cum) > 0.9 were obtained by seven cycles of cross-validation. The closer the parameter values are to 1, the more stable and reliable the model is. After 200 permutation tests, the intercept value of the model was significantly lower than that of the original model, and Q^2^ intercepts were <0, indicating that the established model did not overfit, which further verified that the orthogonal partial least squares discriminant analysis model could effectively distinguish each group of samples. Therefore, the established orthogonal partial least squares discriminant analysis model is stable and reliable and can be used for subsequent difference analysis.

### 3.4. Screening of Significantly Differential Metabolites Among Different Kumquat Germplasms

The number and types of significantly differential metabolites identified in each comparison group are presented in Table 3. From the Table, we found that the differences in metabolites between comparative groups of different kumquat germplasm samples were significant, with a greater number of differentially expressed metabolites showing a downregulated trend. Among them, the fewest differentially expressed metabolites were detected between *F. crassifolia* and *F. margarita*, indicating a high similarity in their metabolites, which is consistent with the PCA results in this study. In contrast, the largest number of differentially expressed metabolites were screened between *F. crassifolia* and *F. hindsii*, indicating a significant difference between the two.

### 3.5. KEGG Pathway Analysis of Differential Metabolites in Different Kumquat Germplasms

#### 3.5.1. Analysis of Differential Metabolite Pathway Between *F. crassifolia* and *F. hindsii* Groups

**Figure 3:**
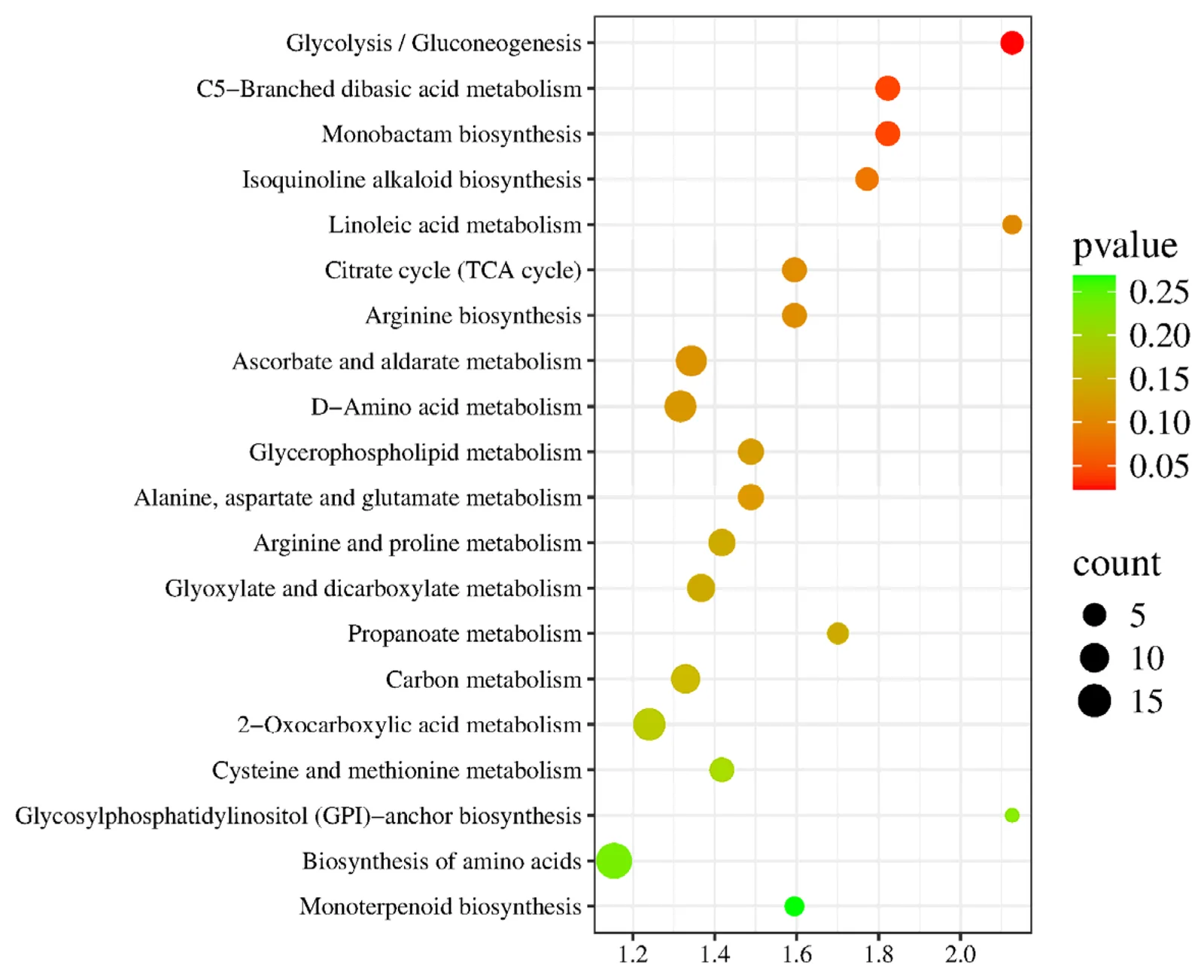
KEGG pathway enrichment map of differential metabolites in the *F. crassifolia* and *F. hindsii* groups.

In the screening of 2500 significantly differential metabolites from the *F. crassifolia* group and the *F. hindsii* groups, 198 metabolites were annotated to 96 metabolic pathways. Based on the enrichment significance and the number of involved metabolites, the top 20 most significantly enriched pathways were selected for visualization, as presented in Figure 3. The comparative analysis revealed five key metabolic pathways exhibiting the most pronounced differential metabolite expression: Glycolysis/Gluconeogenesis, C5-Branched dibasic acid metabolism, Monobactam biosynthesis, Isoquinoline alkaloid biosynthesis, and Linoleic acid metabolism. The Glycolysis/gluconeogenesis pathway demonstrated the highest significance, involving five differential metabolites, including carbohydrates, organic acids, and organic oxides. Notably, while salicin showed significant upregulation, arbutin, glucose, thiamine pyrophosphate, and oxalacetic acid were all significantly downregulated. The C5-branched dibasic acid metabolism pathway, ranking second in significance, contained six acidic differential metabolites (aconitic acid, oxoglutaric acid, (S)-2-acetolactate, etc.), all exhibiting downregulation trends. This pattern indicates higher acid content in *F. crassifolia* compared to *F. hindsii*. In the isoquinoline alkaloid biosynthesis pathway, apart from the upregulation of 4-hydroxyphenylpyruvic acid, other alkaloids, including tyramine and 3,4-dihydroxybenzaldehyde, showed significant downregulation.

#### 3.5.2. Analysis of Differential Metabolite Pathway Between *F. crassifolia* and *F. margarita* Groups

**Figure 4:**
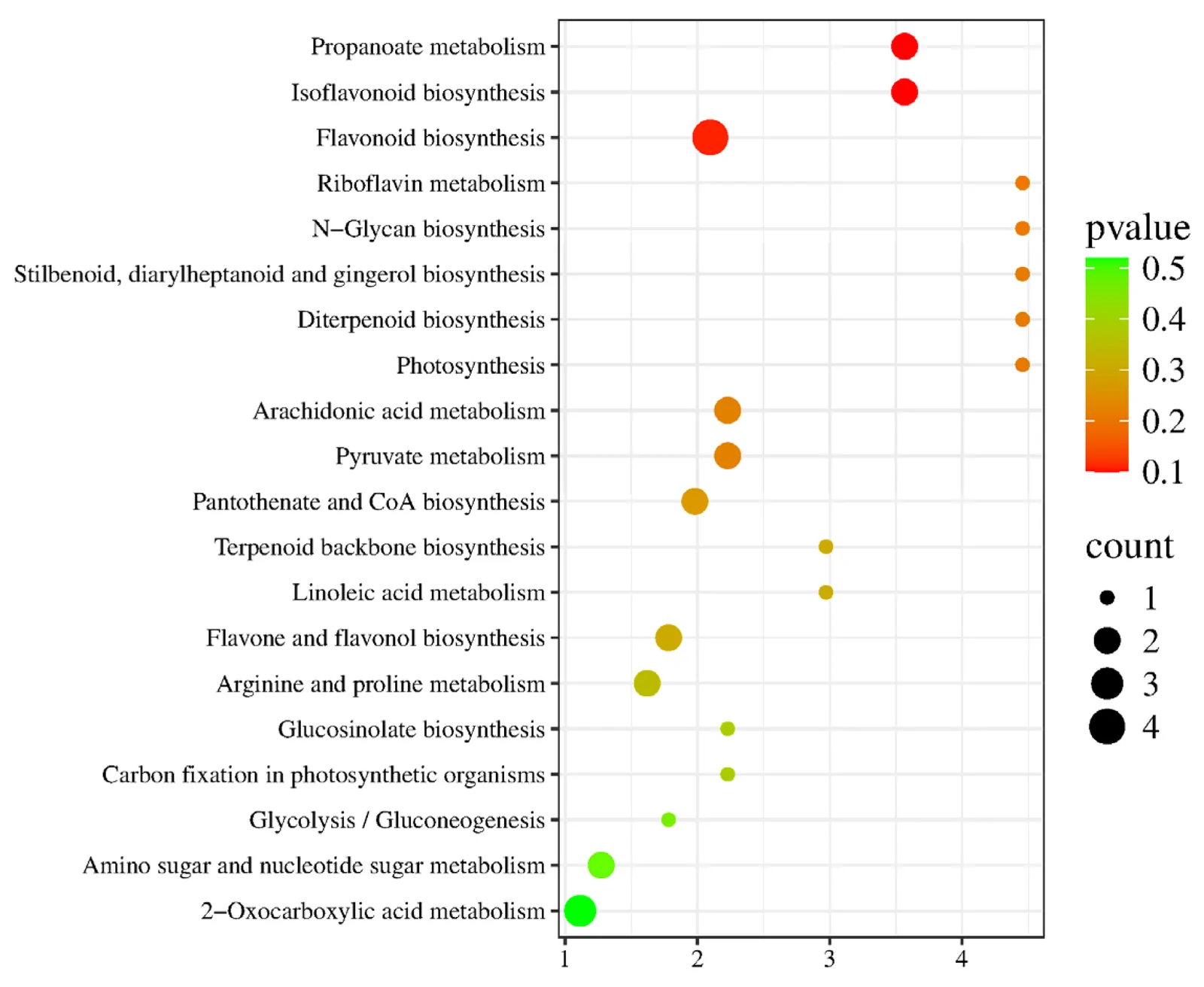
KEGG pathway enrichment map of differential metabolites in the *F. crassifolia* and *F. margarita* groups.

The metabolomics analysis results of the *F. crassifolia* group and the *F. margarita* group showed that a total of 679 significantly differential metabolites were detected, with 47 metabolites annotated to 52 metabolic pathways. The results of metabolic pathway enrichment analysis of the *F. crassifolia* group and the *F. margarita* group (as shown in Figure 4) showed that the five key metabolic pathways with the highest difference in metabolite expression between the *F. crassifolia* group and the F. *margarita* group were propanoate metabolism, isoflavonoid biosynthesis, flavonoid biosynthesis, riboflavin metabolism, and N-Glycan biosynthesis. Among them, the propionic acid metabolic pathway was the most significant, which involved two differential metabolites, methylisocitric acid and (S) -methylmalonic acid semialdehyde, and their relative contents in the *F. margarita* group were significantly upregulated. Four differentially accumulated metabolites were identified in the flavonoid biosynthesis pathway between the two groups, with naringin dihydrochalcone and pinocembrin showing significant upregulation, while 5-O-caffeoylshikimic acid and kaempferol exhibited marked downregulation. Among them, Naringin dihydrochalcone has a sweetness 300 times that of sucrose and can be used as a sugar substitute, helping to reduce human sugar intake and thereby preventing diseases such as obesity and diabetes.

#### 3.5.3. Analysis of Differential Metabolite Pathway Between *F. crassifolia* and *F. obovata* Groups

**Figure 5:**
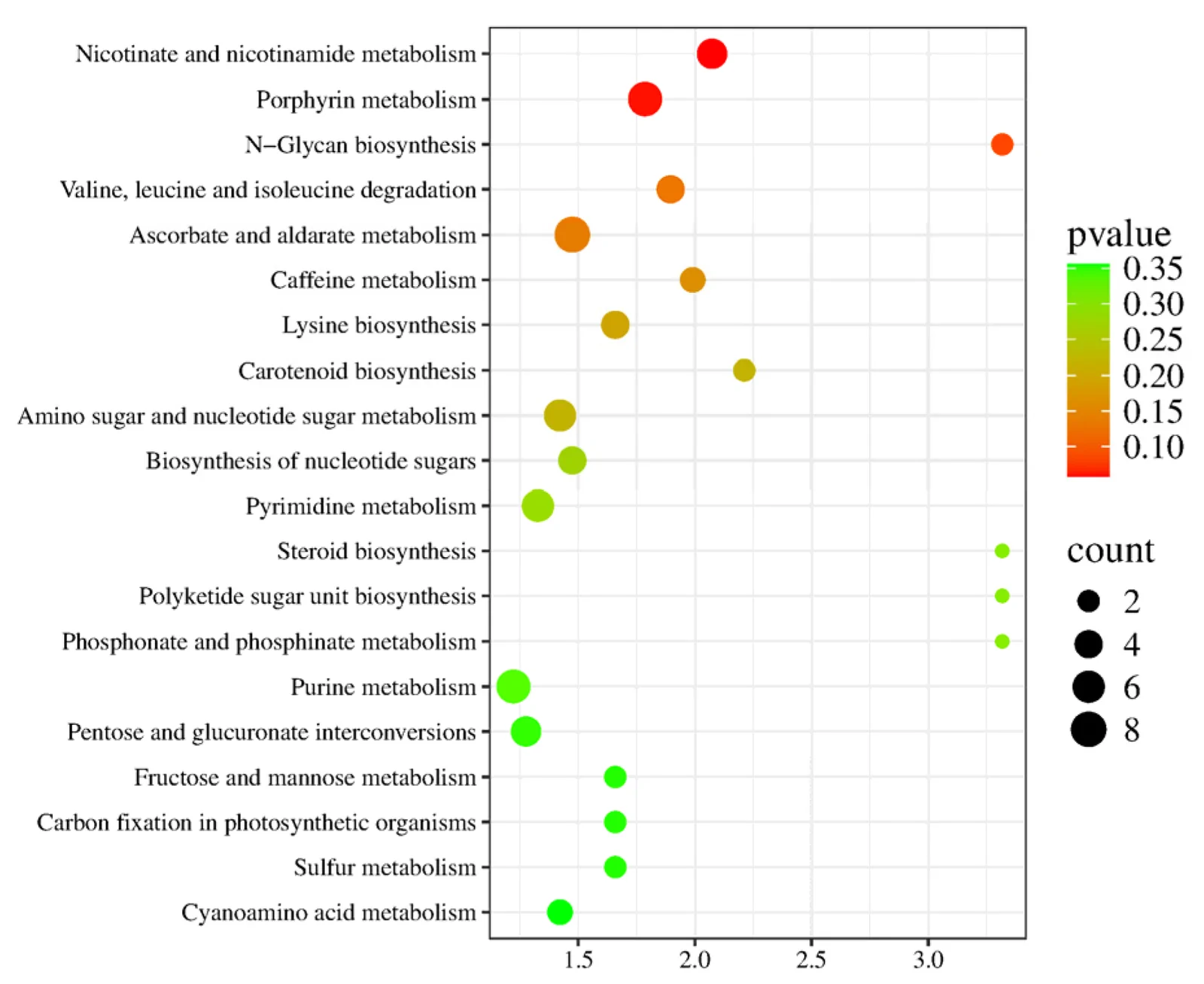
KEGG pathway enrichment map of differential metabolites in the *F. crassifolia* and *F. obovata* groups.

Metabolomic analysis between the *F. crassifolia* group and the *F. obovata* group identified 126 differential metabolites annotated to 80 metabolic pathways. The results of metabolic pathway enrichment analysis of the *F. crassifolia* group and *F. obovata* group are shown in Figure 5. Among them, nicotinate and nicotinamide metabolism, porphyrin metabolism, N-glycan biosynthesis, valine, leucine and isoleucine degradation, and ascorbic acid and aldehyde acid metabolism were the five most significantly differentially expressed metabolic pathways between the two groups. Nicotinate and nicotinamide metabolism was the most significantly enriched pathway, involving five differentially expressed metabolites, such as alkaloids and amino acids. Except for nicotinamide adenine dinucleotide phosphate (NADP^+^), which was significantly downregulated, nicotinamide adenine dinucleotide (NAD), nicotinamide riboside, L-aspartic acid, and trigonelline were all significantly upregulated. This indicates that there are significant differences in the nucleotide metabolism-related pathways between *F. obovata* and *F. crassifolia*. Additionally, the N-glycan biosynthesis pathway showed significant enrichment, with two key metabolites—dolichyl diphosphate+ and dolichyl phosphate D-mannose—being markedly downregulated. These findings suggest that *F. crassifolia* contains higher levels of lipids and saccharide accumulation.

#### 3.5.4. Analysis of Differential Metabolite Pathway Between *F. crassifolia* and *F. japonica* Groups

**Figure 6:**
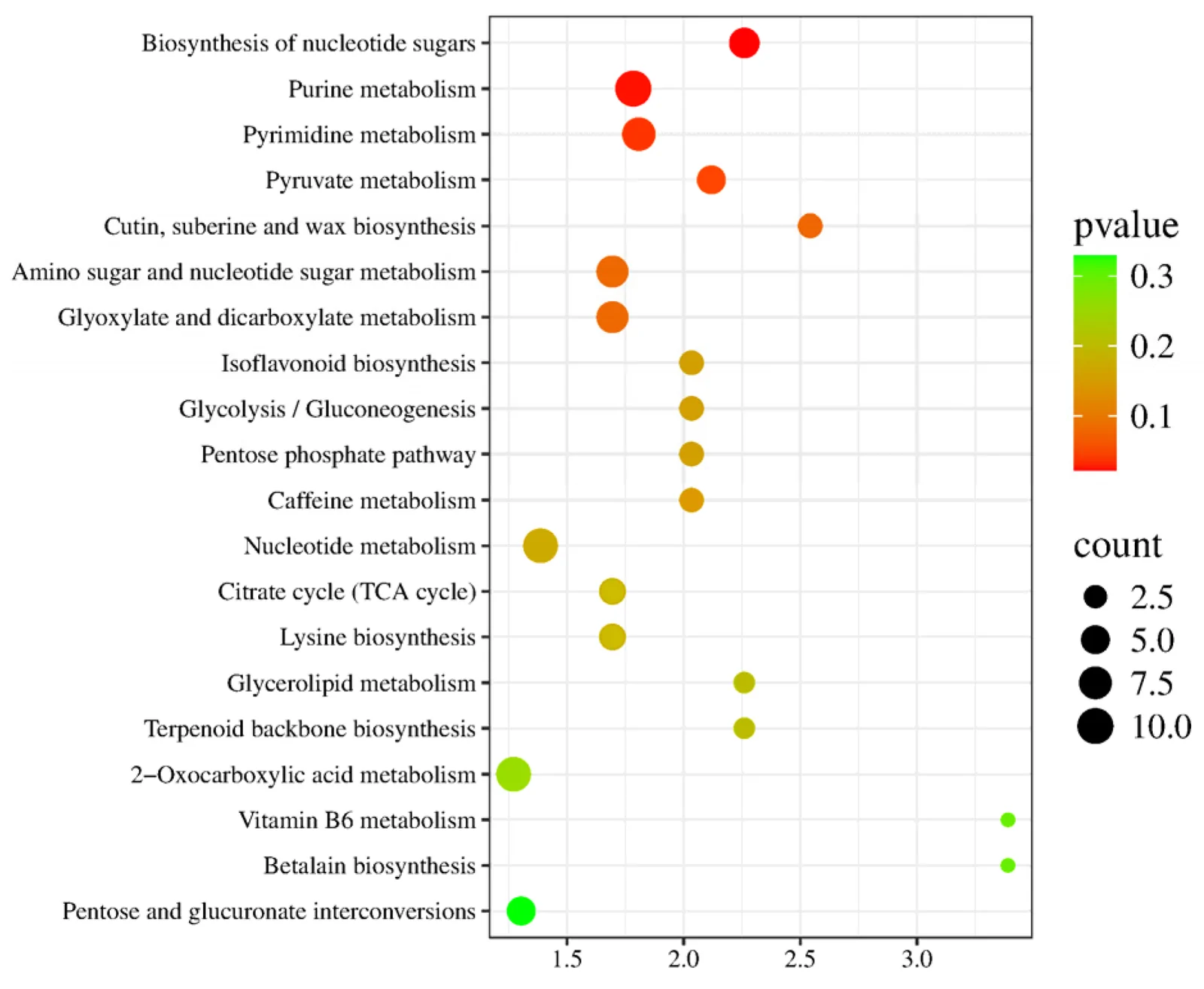
KEGG pathway enrichment map of differential metabolites in the *F. crassifolia* Swingle and *F. japonica* Swingle groups.

In the *F. crassifolia* group and the *F. japonica* group, 123 significantly different metabolites were annotated to 84 metabolic pathways. The metabolic pathway enrichment analysis between the *F. crassifolia* and *F. japonica* groups is presented in Figure 6. Five key metabolic pathways exhibited the most significant differential metabolite expression between these cultivars: (1) Biosynthesis of nucleotide sugars; (2) purine metabolism; (3) pyrimidine metabolism; (4) pyruvate metabolism; and (5) cutin, suberine and wax biosynthesis. Among these, the nucleotide sugar biosynthesis pathway was the most prominent, involving six differentially expressed metabolites, including sugars and organic acids. Notably, with the exception of β-L-fucose 1-phosphate, which showed upregulation, all other metabolites in this pathway demonstrated downregulation trends. In the pyruvate metabolic pathway, five acidic metabolites, including acetyl phosphate and oxaloacetate, showed consistent upregulation, suggesting that the acid content in *F. japonica* may be higher than that in *F. crassifolia*. Regarding the cutin, suberine and wax biosynthesis pathway, three differential metabolites were identified: (S)-10,16-dihydroxyhexadecanoic acid, 16-hydroxyhexadecanoic acid, and floionolic acid, all of which exhibited downregulation trends.

#### 3.5.5. Analysis of Differential Metabolite Pathway Between *F. crassifolia* and Cuipi Groups

**Figure 7:**
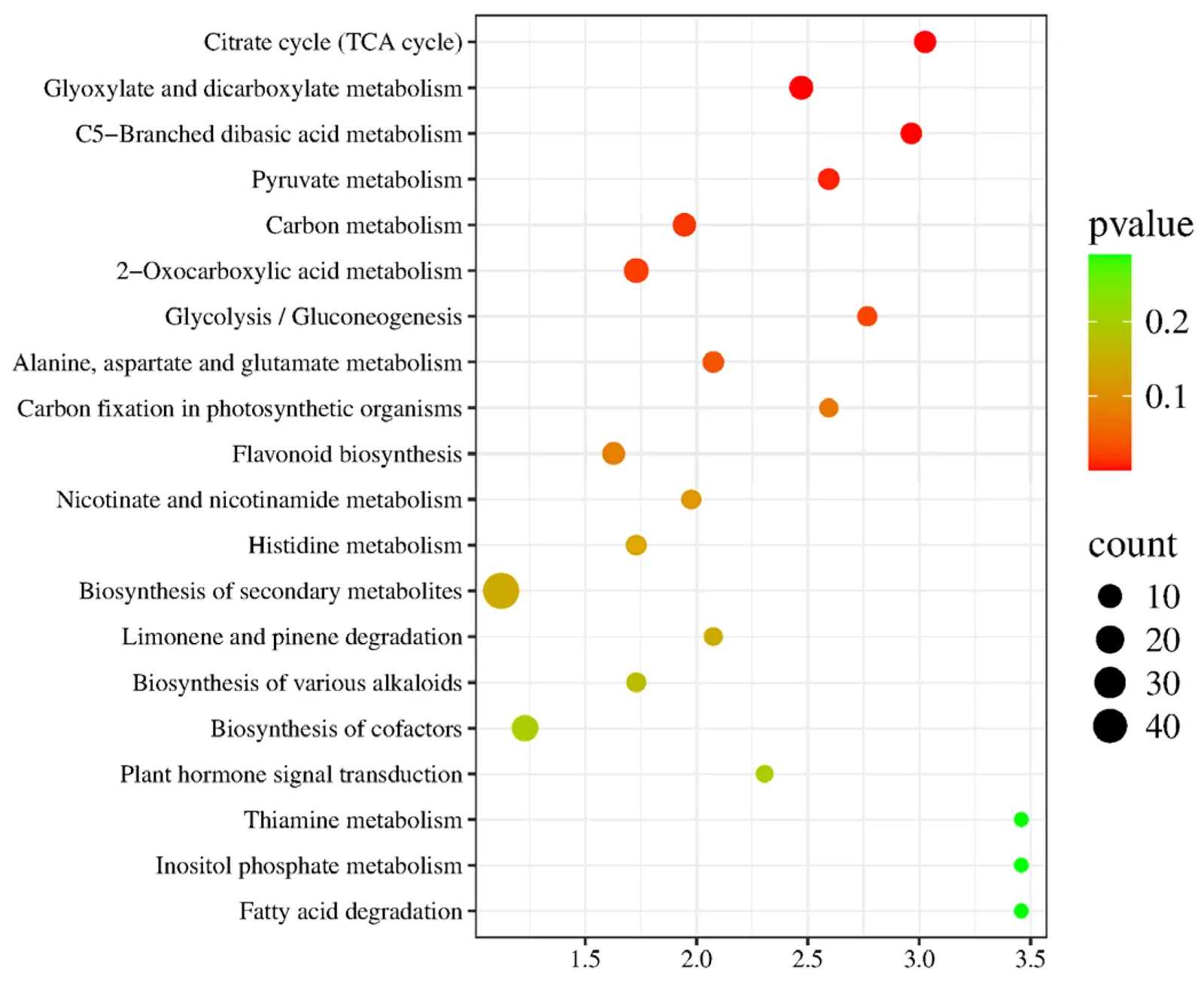
KEGG pathway enrichment map of differential metabolites in the *F. crassifolia* Swingle and Cuipi groups.

A total of 120 significantly differential metabolites were identified between the *F. crassifolia* group and the Cuipi group, which could be annotated to 85 metabolic pathways. Metabolic pathway enrichment analysis between the *F. crassifolia* and Cuipi groups (Figure 7) identified the citrate cycle (TCA cycle) as the most significantly enriched pathway. This pathway involved seven differential metabolites—citric acid, malic acid, isocitrate, oxaloacetate, aconitic acid, and others—all showing distinct downregulation trends. These results indicate more substantial organic acid accumulation in *F. crassifolia*. Furthermore, in the Glyoxylate and dicarboxylate metabolism, C5-branched dibasic acid metabolism, Pyruvate metabolism, and Carbon metabolism pathways, most acidic metabolites—with the exception of glyceric acid and L-aspartic acid—exhibited downregulation trends. This metabolic pattern indicates higher acid content in *F. crassifolia* compared to Cuipi, suggesting that Cuipi possesses superior fresh-eating quality due to its relatively lower acidity.

### 3.6. Analysis of Flavonoid Accumulation Differences in Different Kumquat Germplasms

**Figure 8:**
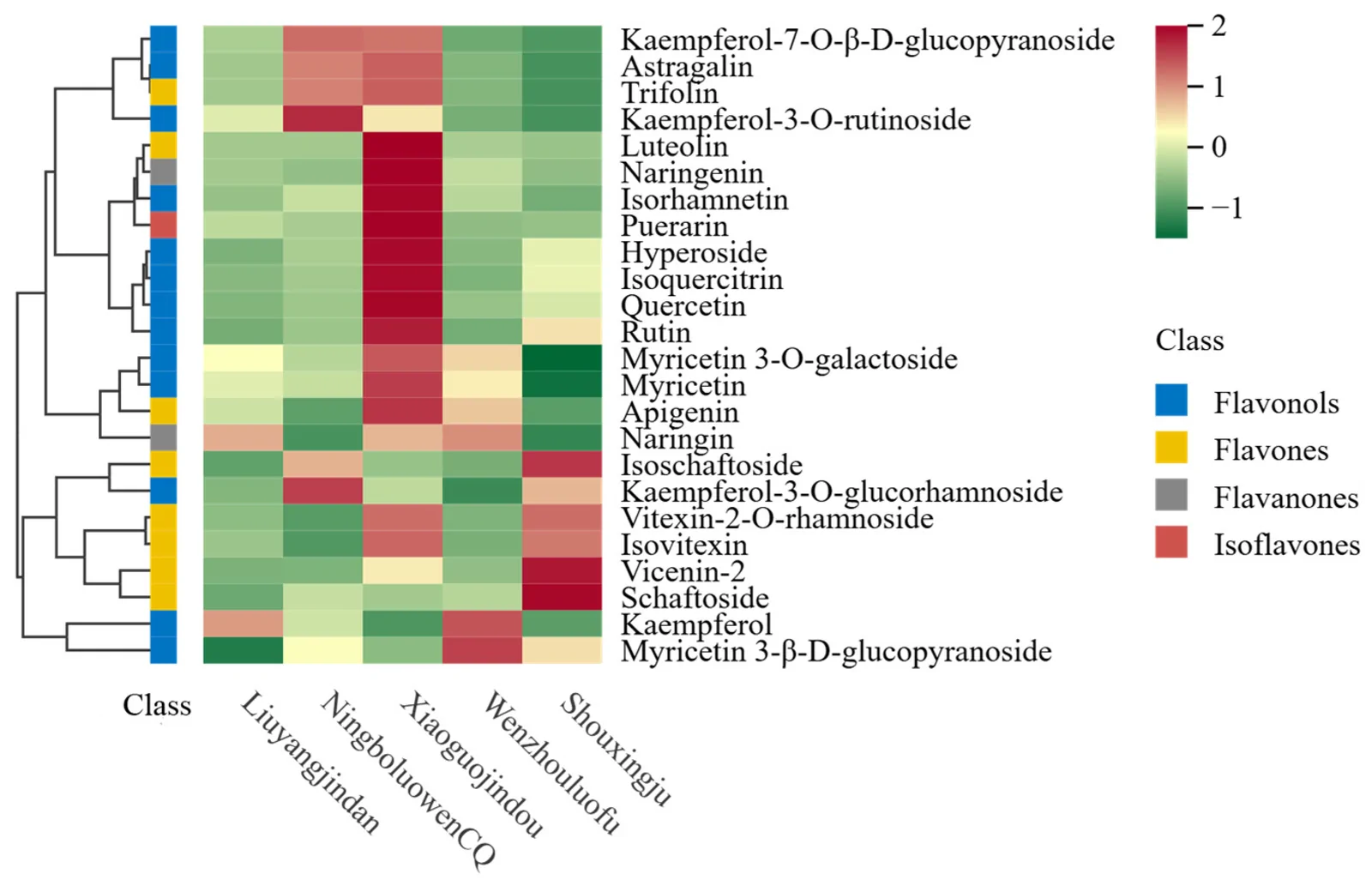
Heatmap analysis of differential flavonoid content in 5 kumquat cultivars.

From the cluster heatmap of 24 flavonoids (Table 4) in five kumquat cultivars (Figure 8), it was observed that the concentrations of 10 flavonoids, including quercetin, hyperoside, and isoquercitrin, were significantly higher in Xiaoguojindou compared to the other cultivars. Additionally, the content of three flavonoids, such as vicenin-2, in Shouxingju showed notable differences from the other kumquats. In NingboluowenCQ, the accumulation of kaempferol-3-O-rutinoside and kaempferol-3-O-glucorhamnoside exhibited significant variations. Wenzhouluofu demonstrated markedly higher levels of myricetin 3-β-D-glucopyranoside and kaempferol than the other cultivars. In contrast, no flavonoids were found to be prominently accumulated in Liuyangjindan compared to the others. These findings indicate that the accumulation of the 24 flavonoids differs significantly among the five kumquat cultivars.

**Figure 9:**
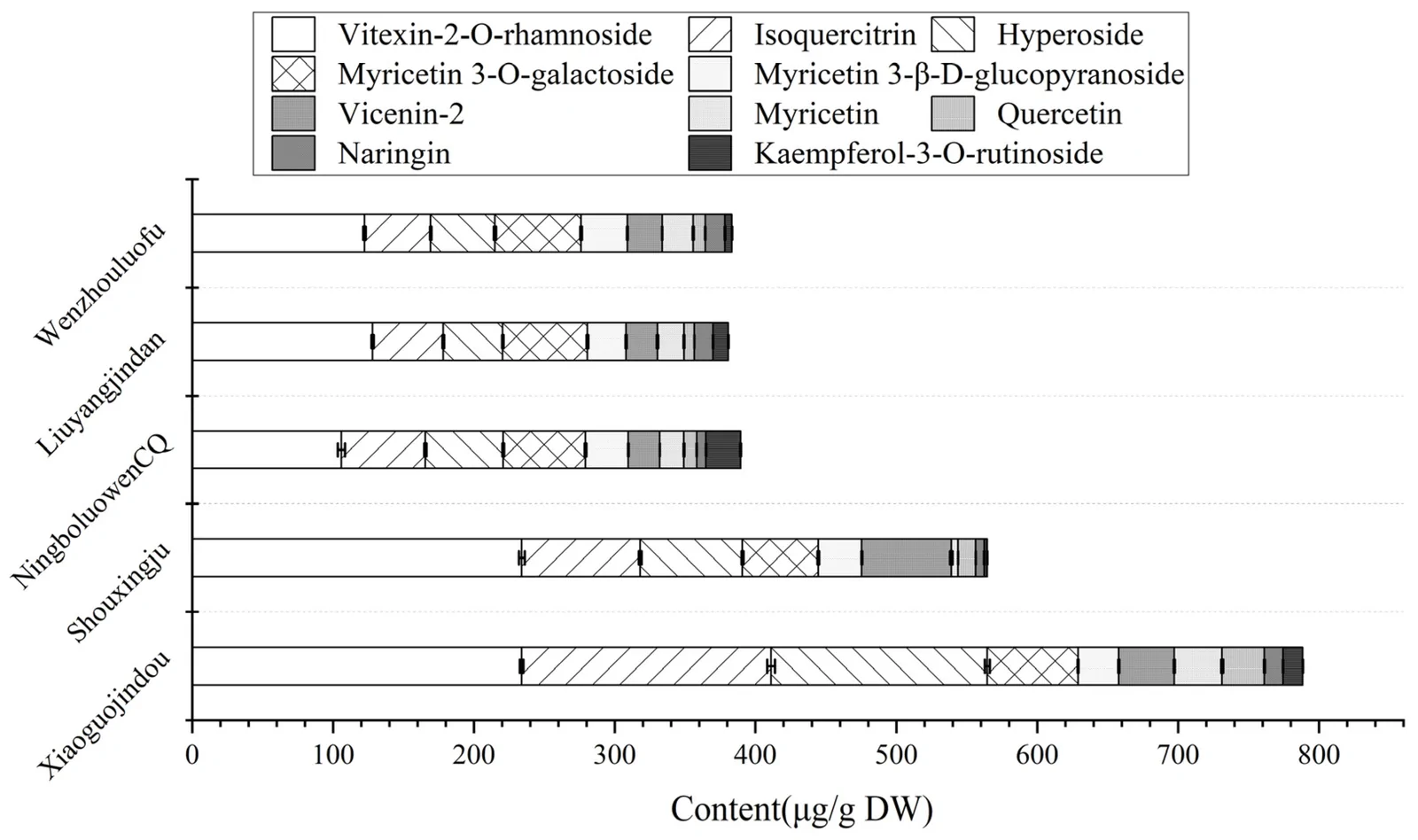
Stacked bar chart of the top 10 flavonoids by content among five kumquat cultivars.

**Figure 10:**
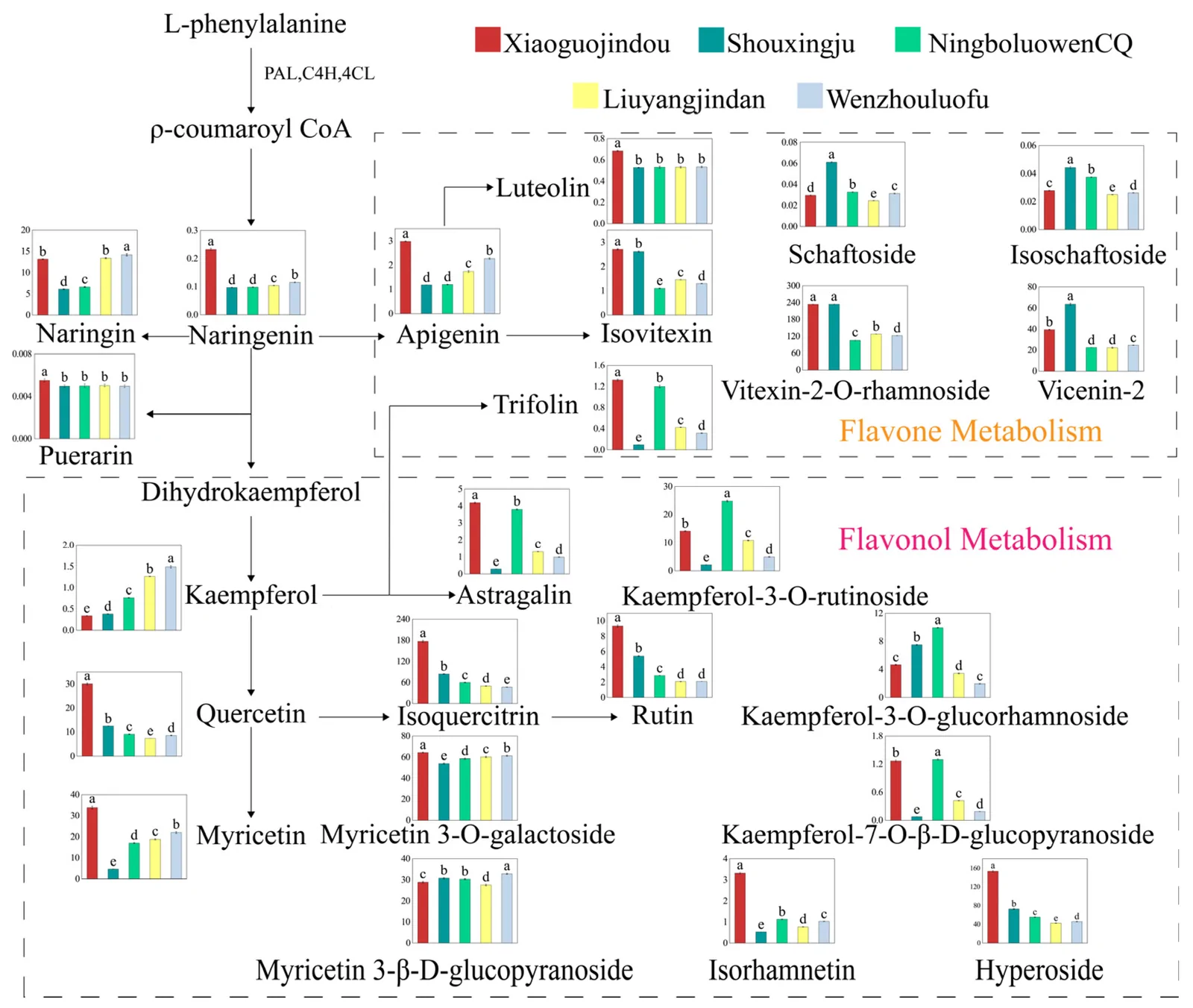
The schematic diagram of the flavonoid metabolic pathway and the histogram of partial metabolite content. Note: For the histogram, different lowercase letters represent statistically significant differences (*p* < 0.05).

Among the 24 flavonoid metabolites measured, ten flavonoids (Figure 9) had an average content exceeding 5 μg/g DW across the five kumquat cultivars. The flavonoid content was highest in Xiaoguojindou, followed by Shouxingju, while the levels in the other three cultivars were relatively similar. Notably, five flavonoids stood out: vitexin-2-O-rhamnoside, isoquercitrin, hyperoside, myricetin 3-O-galactoside, and vicenin-2 (Figure 10). Vitexin-2-O-rhamnoside exhibited the highest content among the five kumquat cultivars. Its content was similar in Shouxingju and Xiaoguojindou, at 233.91 ± 2.02 μg/g DW and 233.82 ± 1.29 μg/g DW, respectively, and was higher than that in the other three cultivars. The lowest content was detected in NingboluowenCQ, at 105.87 ± 2.50 μg/g DW. Vitexin-2-O-rhamnoside was the most abundant among all five cultivars, with similar and higher levels in Shouxingju and Xiaoguojindou (233.91 ± 2.02 μg/g DW and 233.82 ± 1.29 μg/g DW, respectively), while the lowest content was observed in NingboluowenCQ (105.87 ± 2.50 μg/g DW). The concentrations of isoquercitrin and hyperoside were significantly higher in Xiaoguojindou (177.24 ± 2.72 μg/g DW and 153.57 ± 1.78 μg/g DW, respectively) compared to the other four cultivars, which exhibited levels between 40~85 μg/g for these two flavonols. Myricetin 3-O-galactoside content was around 60 μg/g in all five cultivars, with the highest level in Xiaoguojindou (64.35 ± 0.34 μg/g DW). Vicenin-2 was most abundant in Shouxingju (63.60 ± 0.96 μg/g DW), significantly exceeding its levels in the other cultivars. The results indicate that Xiaoguojindou and Shouxingju hold greater potential for medicinal development.

## 4. Discussion

### 4.1. Differential Metabolic Pathways Affect the Quality and Application of Kumquat Fruit

Salicin possesses pharmacological activities such as anti-inflammatory, analgesic, and anti-aging effects, conferring high medicinal value [20]. Researchers have evaluated the antitumor potential of salicin-loaded nanoparticles (SANP) using human MCF-7 cancer cells and found that the resulting SANP and free salicylic acid exhibit anticancer potential in vitro against breast cancer cells, providing a theoretical basis for the development of anti-breast cancer drugs [21]. In the present study, Salicin annotated to the glycolysis/gluconeogenesis pathway was upregulated in *F. hindsii* compared with *F. crassifolia*. This result suggests that *F. hindsii* may possess potential value in the development of Salicin-related functional ingredients.

Nicotinic acid and nicotinamide metabolism was the most significantly differentiated pathway between the *F. crassifolia* group and the *F. obovata* group, and it was more active in *F. obovata*. Nicotinamide riboside in this pathway is a precursor of NAD and a source of vitamin B_3_, and it exhibits functions such as neuroprotection, improving insulin sensitivity, and preventing adverse effects induced by a high-fat diet [22]. Trigonelline is a nitrogen-containing bitter alkaloid with various biological activities, including anti-inflammatory, anticancer, neuroprotective, and antidiabetic effects [23,24]. The upregulation of Trigonelline in *F. obovata* may contribute to a more pronounced bitterness in its fruits, while this finding suggests that *F. obovata* may have potential value for the development of Trigonelline-related functional ingredients.

The nucleotide sugar biosynthesis pathway belongs to the carbohydrate metabolism pathway in plants and plays an important role in protein glycosylation, glycogen biosynthesis, and even cell wall synthesis [25]. In this study, the nucleotide sugar biosynthesis pathway showed significant differences between *F. crassifolia* and *F. japonica*, in which UDP-D-Galactose was upregulated in *F. japonica*. UDP-D-Galactose is generated from UDP-D-Glucose catalyzed by UDP-D-glucose 4-epimerase (UGE4). By regulating the supply of UDP-D-Galactose, UGE4 can indirectly affect the synthesis of cell wall polysaccharides [26]. Therefore, the upregulation of UDP-D-Galactose in *F. japonica* may influence its flesh texture.

Organic acids not only affect the nutritional value of fruits but also serve as a major factor influencing fruit flavor [27]. Most fruits accumulate various organic acids during their ripening stage. Based on the types of organic acids accumulated, they can be classified into citric acid-accumulating type, tartaric acid-accumulating type, malic acid-accumulating type, etc. Citrus fruits are typical citric acid-accumulating types [28,29]. In this study, compared with *F. crassifolia*, five acidic metabolites in the pyruvate metabolism pathway of *F. japonica*, including acetyl phosphate and oxalacetic acid, were found to be downregulated. Additionally, seven acidic metabolites in the TCA cycle of Cuipi, including citric acid and malic acid, were also downregulated. This indicates that the relative content of acidic metabolites in Cuipi is lower than that in *F. crassifolia* and *F. margarita*. Yang [2] et al. previously reported that the titratable acidity of the two kumquat germplasms in the Cuipi group was significantly lower than that of other kumquat germplasms, which is consistent with the analysis results of acid metabolites in the present study. Therefore, it is speculated that the kumquats in the Cuipi group are more suitable for fresh consumption.

Through untargeted metabolomics analysis, this study identified significant differential metabolites between *F. crassifolia* and other kumquat germplasms. These differential metabolites may not only reflect changes in related metabolic pathways but also serve as the metabolic basis for the differences in fruit phenotypes and functional components among different germplasms. The relative contents of several major organic acids were lower in Cuipi, suggesting that it is more suitable for fresh consumption. Furthermore, considering the pharmacological activities of these differential metabolites reported in the literature, future studies can further validate the pharmacological effects of characteristic metabolites in the fruits of different kumquat germplasms (such as Salicin in *F. hindsii*, Nicotinamide Riboside, and Trigonelline in *F. obovata*), and optimize their extraction processes, thereby providing a scientific basis for the precise development and utilization of different kumquat germplasms.

### 4.2. The Difference in Flavonoid Accumulation Affects the Production and Application of Kumquat Fruit

The analysis of 24 flavonoids in the text revealed significant differences in their distribution among five cultivars of kumquats, providing guidance for the production and application of these kumquat fruits. Vitexin-2-O-Rhamnoside was one of the relatively abundant flavonoids in the fruits of the five kumquat species, which is consistent with the findings of Guo [14] et al. Furthermore, the present study revealed that this compound accumulated at higher levels in Xiaoguojindou and Shouxingju. Studies have shown that this compound possesses various biological activities, such as anti-inflammatory and anti-atherosclerotic effects [30], as well as immunomodulatory properties [31]. Therefore, Xiaoguojindou and Shouxingju can be utilized for the development and extraction of vitexin-2-O-rhamnoside. As the germplasm with the highest flavonoid content among the five kumquat cultivars, *F. hindsii* accumulates high levels of quercetin, isoquercitrin, hyperoside, and myricetin in its fruits, in addition to vitexin-2-O-rhamnoside. Studies have shown that the four metabolites kaempferol, quercetin, isorhamnetin, and myricetin constitute the four common flavonol aglycones in citrus, which subsequently form stable flavonol glycosides in citrus through a series of glycosyltransferase reactions [32]. Moreover, these flavonols exhibit pharmacological effects, such as anticancer and antitumor [33,34], antibacterial [35], and antioxidant [36] effects. Among them, quercetin shows outstanding effects in rheumatoid arthritis [37], myocardial protection [38], and spinal cord repair [39]. Therefore, functional validation of these metabolites in Xiaoguojindou can be conducted to explore their potential for medicinal development. Naringin is a flavanone abundantly found in pomelo and citrus fruits [40,41] and is a major contributor to the bitterness of these fruits. In this study, the naringin content in the five kumquat cultivars was measured to be between 6.081 and 14.196 μg/g, which was similar to the content of naringin in kumquat determined by Wang et al. [42], and it was far lower than the content of naringin in pomelo. This may be one of the reasons for the high edibility of kumquat fruits.

In summary, the total flavonoid content in Xiaoguojindou was significantly higher than that in the other four kumquat species, and most flavonoids were more abundant in Xiaoguojindou. Moreover, Xiaoguojindou contains very little pulp, and its peel is easily separated from the seeds; therefore, compared with the other four species, it has greater potential for medicinal development. Although only one germplasm from each of the five kumquat species was selected for quantitative flavonoid analysis in this section—which cannot fully represent the flavonoid profiles of all germplasms within these five species—the results obtained from these five germplasms can still provide a solid basis for the development and utilization of flavonoids in the fruits of these five kumquat species.

## 5. Conclusions

In summary, by integrating untargeted and targeted metabolomics, this study systematically revealed the metabolic differences and development potential among different kumquat germplasms. Based on this analysis, *F. crassifolia* and Cuipi are more suitable for fresh consumption. Future studies can focus on characteristic metabolites, such as salicin and flavonoids in *F. hindsii*, and nicotinamide riboside and trigonelline in *F. obovata*, to conduct pharmacological validation and optimize extraction processes, thereby further exploring their potential for medicinal development. Meanwhile, future research should perform more comprehensive targeted quantitative analyses of metabolites such as flavonoids and phenolic acids in kumquat germplasms. Combined with genomics and other omics approaches to further elucidate the key metabolic pathways, this will provide a more solid theoretical foundation for variety breeding, processing applications, and functional component development of kumquats, ultimately promoting the high-quality development of the kumquat industry.

## Figures and Tables

**Table 1:** Information of kumquat samples.

Number	Cultivar	Species Name	Origin
1	Wenzhouluofu	*F. margarita* Swingle	Wenzhou, Zhejiang, China
2	LiuyangjinganCQ	*F. crassifolia* Swingle	Liuyang, Hunan, China
3	WenzhoujindanCS	*F. crassifolia* Swingle	Wenzhou, Zhejiang, China
4	Shouxingju	*F. obovata* Tanaka	Sichuang, China
5	Daguoluofu	*F. margarita* Swingle	Yongchun, Fujian, China
6	NingboluowenCQ	*F. japonica* Swingle	Ningbo, Zhejiang, China
7	Liuyangjindan	*F. crassifolia* Swingle	Liuyang, Hunan, China
8	Lanshanjingan	*F. crassifolia* Swingle	Lanshan, Hunan, China
9	Xiaoguojindou	*F. hindsii* Swingle	Chenzhou, Hunan, China
10	Daguojindou	*F. hindsii* Swingle	Chenzhou, Hunan, China
11	Huapijingan	*F. crassifolia* Swingle(cuipi)	Yangshuo, Guangxi, China
12	Rong’ancuimi	*F. crassifolia* Swingle(cuipi)	Rong’an, Guangxi, China
13	Suichuanjindan	*F. crassifolia* Swingle	Suichuan, Jiangxi, China

Note: The letters in Table 1 germplasm represent the abbreviation of the introduced city, namely CQ, Chongqing (Citrus Research Institute, Chinese Academy of Agricultural Sciences); CS, Changsha (Hunan Horticultural Research Institute).

**Table 2:** Evaluation parameters of OPLS-DA model.

Comparison Group	Positive Ion Mode						Negative Ion Mode					
	A	R^2^X (cum)	R^2^Y (cum)	Q^2^ (cum)	R^2^ Intercepts	Q^2^ Intercepts	A	R^2^X (cum)	R^2^Y (cum)	Q^2^ (cum)	R^2^ Intercepts	Q^2^ Intercepts
*F. crassifolia* Swingle vs. *F. hindsii* Swingle	3	0.871	1	1	0.238	−0.729	3	0.879	1	1	0.23	−0.731
*F. crassifolia* Swingle vs. *F. margarita* Swingle	5	0.892	0.999	0.998	0.273	−1.1	5	0.886	1	1	0.28	−0.949
*F. crassifolia* Swingle vs. *F. obovata* Tanaka	3	0.809	1	0.999	0.218	0.992	3	0.809	0.999	0.998	0.174	−0.948
*F. crassifolia* Swingle vs. *F. japonica* Swingle	3	0.785	0.999	0.998	0.213	−0.949	3	0.579	1	0.996	0.182	−0.999
*F. crassifolia* Swingle vs. *F. crassifolia* Swingle (Cuipi)	3	0.879	0.998	0.998	0.22	−0.763	3	0.863	0.998	0.997	0.217	−0.737

Note: A represents the number of principal components of the model; R^2^X (cum) represents the explanatory power of the model for X variables; R^2^Y (cum) represents the explanatory power of the model for the Y variable; Q^2^ (cum) represents the predictability of the model; R^2^ intercepts and Q^2^ intercepts represent the intercepts of R^2^ and Q^2^ regression lines for the Y axis.

**Table 3:** Number and variation in differential metabolites screened by different kumquat germplasms.

Comparison Group	Number of Differential Metabolites	Variation in Differential Metabolites	
		Upregulated Trend	Downregulated Trend
*F. crassifolia* Swingle vs. *F. hindsii* Swingle	2500	1017	1483
*F. crassifolia* Swingle vs. *F. margarita* Swingle	679	293	386
*F. crassifolia* Swingle vs. *F. obovata* Tanaka	1703	670	1033
*F. crassifolia* Swingle vs. *F. japonica* Swingle	1495	690	805
*F. crassifolia* Swingle vs. *F. crassifolia* Swingle(Cuipi)	1792	627	1165

Note: The downregulated trend of differential metabolite labeling was higher in *F. crassifolia* Swingle, and the upregulated trend was higher in other kumquat germplasms.

**Table 4:** 24 kinds of flavonoids and their classification.

Class	Metabolites	Metabolites
Flavones	Vitexin-2-O-rhamnoside	Luteolin
	Vicenin-2	Isoschaftoside
	Isovitexin	Schaftoside
	Trifolin	Apigenin
Flavonols	Myricetin	Quercetin
	Myricetin 3-O-galactoside	Isoquercitrin
	Myricetin 3-β-D-glucopyranoside	Hyperoside
	Kaempferol	Isorhamnetin
	Kaempferol-3-O-rutinoside	Rutin,
	Kaempferol-3-O-glucorhamnoside	Astragalin
	Kaempferol-7-O-β-D-glucopyranoside	
Flavanones	Naringenin	Naringin
Isoflavones	Puerarin	

## Data Availability

The original contributions presented in this study are included in the article. Further inquiries can be directed to the corresponding author.
